# Claudin and pancreatic cancer

**DOI:** 10.3389/fonc.2023.1136227

**Published:** 2023-03-07

**Authors:** Chen Wang, Na Wu, Beibei Pei, Xiaoyan Ma, Wenhui Yang

**Affiliations:** ^1^ Shanxi Medical University, Taiyuan, Shanxi, China; ^2^ Department of Gastroenterology, Shanxi Province Cancer Hospital/ Shanxi Hospital Affiliated to Cancer Hospital, Chinese Academy of Medical Sciences/Cancer Hospital Affiliated to Shanxi Medical University, Taiyuan, China; ^3^ Third Hospital of Shanxi Medical University, Shanxi Bethune Hospital, Shanxi Academy of Medical Sciences, Tongji Shanxi Hospital, Taiyuan, China

**Keywords:** pancreatic cancer (PCA), claudin (CLDN), diagnosis, targeted therapy, claudin-18.2, claudin-4

## Abstract

Due to the lack of timely and accurate screening modalities and treatments, most pancreatic cancer (PCa) patients undergo fatal PCa progression within a short period since diagnosis. The claudin(CLDN) family is expressed specifically as tight junction structure in a variety of tumors, including PCa, and affects tumor progression by changing the cell junctions. Thus far, many of the 27 members of the claudin family, including claudin-18.2 and claudin-4, have significantly aberrantly expression in pancreatic tumors. In addition, some studies have confirmed the role of some claudin proteins in the diagnosis and treatment of pancreatic tumors. By targeting different targets of claudin protein and combining chemotherapy, further enhance tumor cell necrosis and inhibit tumor invasion and metastasis. Claudins can either promote or inhibit the development of pancreatic cancer, which indicates that the diagnosis and treatment of different kinds of claudins require to consider different biological characteristics. This literature summarizes the functional characteristics and clinical applications of various claudin proteins in Pca cells, with a focus on claudin-18.2 and claudin-4.

## 1 Introduction

Pancreatic cancer (PCa) is one of the most common cancers in the world and the seventh leading cause of cancer-related deaths worldwide. PCa incidence is high in countries with high human development index and continues to increase yearly (1). PCa is one of the most malignant diseases in humans owing to its high invasive ability, frequent metastasis, and recurrence. Furthermore, due to the low rate of early diagnosis, rapid disease progression, and poor prognosis,most PCa patients are already in the middle or late stages of cancer when diagnosed. Additionally, the development of local lymphatic infiltration or distant metastasis in these stages makes surgical treatment ineffective, making the median survival <6 months and the 5-year survival rate <5.5% (2, 3). PCa primarily originates from the malignant transformation of cells in the pancreatic ductal epithelium and alveolar cells, and its subtypes are divided into pancreatic ductal adenocarcinoma (PDAC), alveolar carcinoma, pancreatic blastoma, and neuroendocrine tumors, with PDAC accounting for 90% of the PCa cases.

PCa metastasis mainly occurs in the liver, lungs, and bones. Epithelial–mesenchymal transition (EMT) is an important step leading to the invasion and migration of various tumor cells (4). EMT first occurs with contact rupture between epithelial cells, such as tight junctions (TJs), bridging granules, gap junctions, and adhesion junctions, ultimately leading to changes in cell polarity, cytoskeletal reorganization, and loss of tumor cell metastasis (5). Claudin (CLDN) proteins are members of TJs and almost participated in all steps of tumor development (6). In recent years, claudin has been increasingly studied, and many claudin proteins, such as claudin-18.2, claudin-4, and claudin-7 have been demonstrated to be ideal targets for tumor therapy. Moreover, several claudin-targeting drugs for PCa, gastric cancer (GC), and colon cancer treatment are already in the clinical trial stage. This review aims to summarize the mechanism of claudins in the occurrence, development and metastasis of pancreatic cancer, as well as the prospect and application of claudins in the diagnosis and treatment of pancreatic cancer.

## 2 Claudin protein structure and regulation

**Figure 1:**
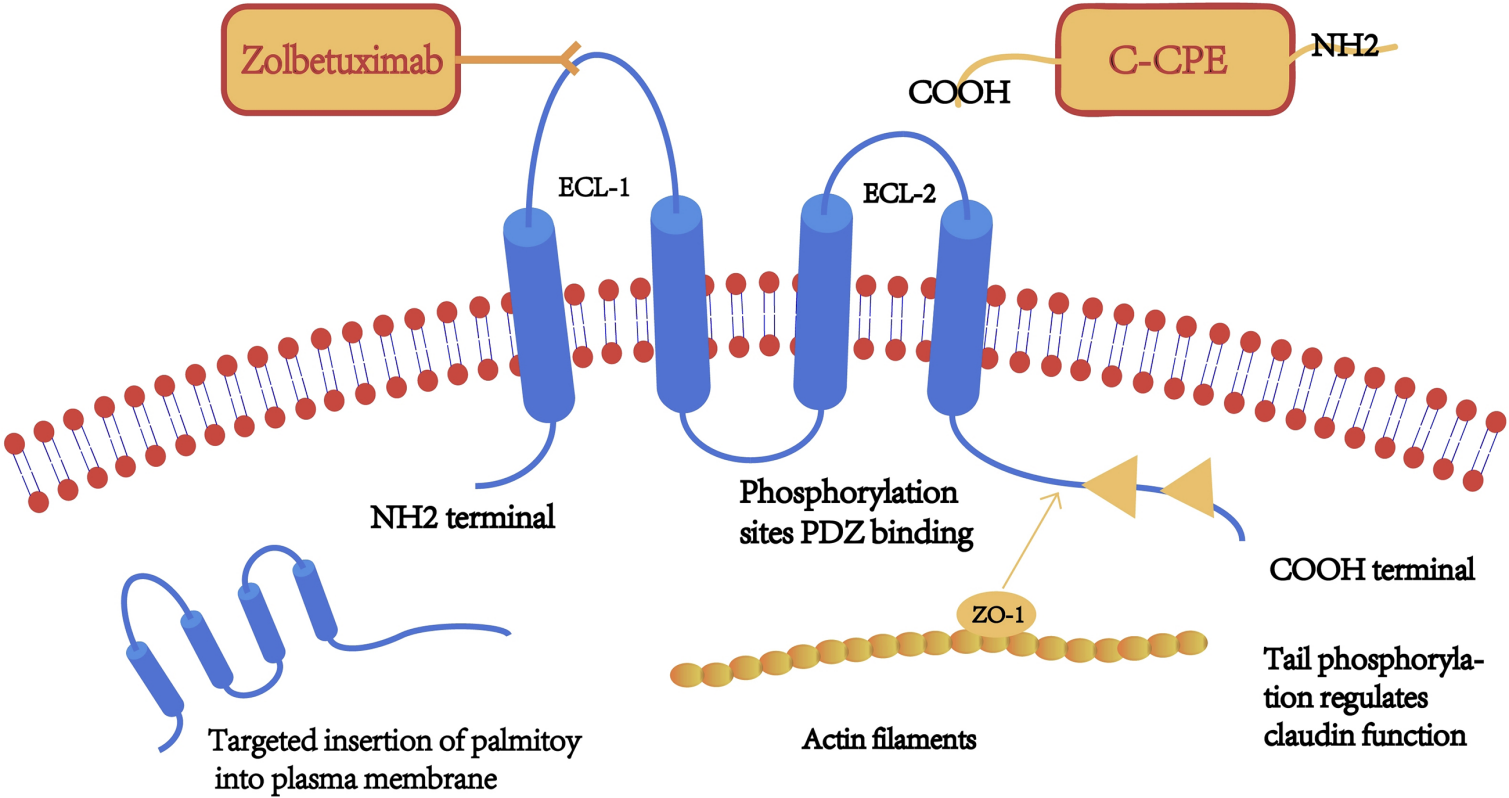
A schematic diagram of the claudin (CLDN) protein structure. Claudin has four transmembrane structures, a highly conserved extracellular loop (ECL)-1 sequence, a monoclonal antibody binding site (claudin-18.2), and serine/threonine/tyrosine kinase binding sites on the C-terminal. Claudin tail is inserted into the cell membrane after palmitoylation.

Claudins are a family of transmembrane proteins with a molecular size of 17–27 kDa. The 27 Claudin family genes are known to be located at chromosome 3q22, 7q11, 7q11, etc (7), approximately 35 kb, consisting of six exons and five introns. The *CLDN* gene encodes tetraspanin, a key structural and functional component of TJs that forms a paracellular barrier. It controls the size of the paracellular space through which molecules pass in the epithelial and endothelial tissues (8), thereby controlling the flow of molecules between the cells. The claudin family plays a key role in the carcinogenesis of related tissues (9). Claudin was first identified and reported by Mikio et al. in a mouse protein screen, in which it was found to interact with the known intact TJ protein, occludin (10). Claudin and ocludin together form the TJ structure. Claudin has 4 transmembrane structural domains, with the N- and C-terminals on the cytoplasmic side, the phosphorylation site located on the C-terminal, and 2 extracellular loops (ECL), located on the N-terminal 28–76 aa (ECL1) and 141–159 aa (ECL2) (11). ECL1 contains charged amino acids and is arranged in the cellular bypass, thus it determines the charge selectivity of paracellular transport. In contrast, ECL2 acts as the binding site for some targeting molecules, such as the C-terminal of Clostridium perfringens enterotoxin (C-CPE) molecules. The C-terminus interacts with cytoplasmic scaffolds ZO-1, ZO-2 and ZO-3 *via* PDZ binding motifs,and interacts with the first PDZ structural domains of cytoplasmic scaffolds ZO-1 and ZO-2 interact to form the TJ chain and play a key role in epithelial barrier formation (12). Simultaneous deletion of claudins and the TJ membrane protein JAM-A resulted in loss of membrane attachment and macromolecular permeation barriers and led to an episodic epithelial polarity defect. These results suggest that claudins and JAM-A synergistically regulate TJ formation and epithelial polarity (13). Furthermore, claudin proteins are distinguished by the structural heterogeneity of the C-terminal tail, which contributes to the heterodimer-dependent paracellular selectivity function through differences in targeting and regulation by different proteins and signaling molecules. The localization and function of claudin are also regulated by the phosphorylation of the C-terminal, a target of serine, threonine, and tyrosine kinases. (**Figure 1**) Claudin regulates the functional changes of TJ protein through post-translational modifications. Post-translational modifications such as phosphorylation, ubiquitination, palmitoylation and glycosylation are processes that affect claudin conformation, stability, transport and function.

In the study of claudin, phosphorylation and palmitoylation are the more studied modifications (14). Natural claudins are palmitoylated almost exclusively in the intracellular loop and helix 4. Claudin-3 is phosphorylated on Thr-192, claudin-4 at Ser-194, Thr189, claudin-5 at Thr-205 and Thr-207, claudin-16 at Ser-217 (15). Much MS data14 identifies phosphorylation of Y(-1) in claudin1-7, -9, -10 and claudin-18. Claudin-4 at the -1 position decreases binding to ZO-1 (16, 17). Phosphorylation of claudin is associated with the maintenance of normal barrier function in TJs, while dephosphorylation produces a negative regulatory effect (18, 19). In cancer, phosphorylation of most claudins disrupts the function of TJs (19). Palmitoylation in post-translational modifications of claudin is necessary for the targeting and anchoring of certain claudin proteins to the cell membrane and for efficient insertion of TJ, and four dominant sites exist for palmitoylation of the tail end of claudin after palmitoylation into the cell membrane claudin-3, -4, -6, the addition of C-CPE does not immediately alter palmitoylation of claudin-4, and it was found that these claudin also contains several highly occupied palmitoylation sites. In classical claudin palmitoylation of cysteines is thought to occur only in the intracellular loops of tightly attached flanks and in helix 4; recent studies have found that claudin can be palmitoylated in all transmembrane helices by cysteine scanning. Palmitoylation on other helices may interfere with claudin function, for example, palmitic acid attached to helix 1 may prevent the interaction required for claudin-claudin to form a tight junction. This is a step necessary for the targeting and anchoring of some types of claudin to the cell membrane and the effective insertion of tight junctions (20, 21). Aberrant expression of *CLDN* in cancer occurs during and after transcriptional regulation of transcription factors as well as epigenetic regulation of DNA methylation, histone modifications, and miRNAs (22). Snails (a transcription factor that plays a key role in the EMT process) represses the expression of *CLDN*1,3,4and7 as well as E-cadherin by directly binding to the promoter of *CLDN*.

Claudin is generally located at the apical of the cell, thus maintaining cell polarity. When tissues undergo pathological changes, claudin can be undergo phosphorylation, leading to a change in cell polarity. Claudins have different roles in different tumor tissues, and the same claudin protein can be differentially expressed in the tumor and normal tissues of the same organ. Although the expression pattern of claudin is tissue-specific, most tissues express multiple claudins simultaneously, which interact in a homotypic and heterotypic manner to form tightly connected intercellular structures (23). Several studies have been conducted on the structural properties of claudin, and claudin-18.2 and claudin-4 have been demonstrated to be ideal therapeutic targets for PCa treatment, with the claudin -18.2- and claudin-4-targeted drugs in the clinical trial stages.

## 3 Dysregulated expression of CLDNs in cancer

The adjacent cells interact using the claudin proteins to form adhesion structures, and the composition of claudin proteins in a given tissue alters the selectivity and strength of TJs, thereby altering the strength and function of the tissue junctions (24). *CLDN* overexpression or downregulation in tumor cells promotes tumor infiltration, growth, and metastasis. Moreover, the same type of claudin produces different effects in different tumor types. For instance, claudin-4 expression is upregulated with disease progression in PCa, colorectal cancer (CRC), esophageal squamous cell carcinoma, and GC tissues, and its upregulation is associated with reduced invasiveness and metastatic potential, which is positively correlated with better prognosis. In contrast, *CLDN 4* overexpression in breast cancer (BC), lung cancer (LC), ovarian cancer (OC), and PCa is positively correlated with invasion, metastasis, angiogenesis, and poor prognosis. Claudin proteins are extensively studied, and various claudin proteins, including claudin-1, claudin-4, and claudin-18.2 have high specificity and sensitivity in tumor diagnosis (25). Claudin-11 plays an important role in nerve infiltration in PCa, with claudin-11 expression suppressed during nerve invasion and overexpressed during the low incidence of nerve infiltration (26). Claudin is distributed in both normal epithelial cells and cancer cells, and a dyslocalization of claudin occurs from the tight junctions to the cell surface, when the tissue is malignantly altered, in contrast to normal conditions where claudin is localized in the tight junctions between cells. Therefore, the heterogeneity and differential expression of claudin proteins in different tissues, cancers, and disease stages make claudins potential diagnostic markers for the early detection of different cancers and specific prognostic indicators for cancer classification (27) (**Table 1**).

**Table 1:** Claudin (CLDN) protein functions, clinical significance, and the associated signaling.

Claudin type	Main functions and clinical significance in PCa	Signaling molecules involved	Citatio-ns
claudin-1	• normal expression maintains the normal function of cells • decreased expression promotes tumor cell invasion and metastasis	• ZEB1-regulated overexpression of ZIP4 reduces the expression of ZO-1 and claudin-1, leading to the phosphorylation of FAK and Paxillin, thus promoting PCa invasion and migration • 5-HT1B, 5-HT1D, and eEF2K/MAPK-Erk pathway	(28–33)
claudin-2	• decreased expression with an increase in tumor histological grade • decreased expression in exocrine adenocarcinoma, cystic myxoma, and acinar cell carcinoma	• miR-222-3p and YAP	(33–36)
claudin-4	• differentially expressed in PCa • effective inhibitor of the invasive and metastatic phenotype of PCa cells	• Ras/Raf/Erk pathway • TGF-β and DEC1 siRNA	(37–39)
claudin-5	• maintains cell polarity and cell barrier • vascular endothelium, lymphatic vessels, and tumor interstitial blood vessel	• CD99	(40–42)
claudin-6	• expression in embryonic stem cells • no expression in normal adult tissues • promotes chemotherapy resistance of MCF-7 cells	• regulates GSTP1 • promotes the chemotherapy resistance of MCF-7 cells *via* p53	(14, 43–46)
claudin-7	• decrease in expression is parallel to the degree of tumor differentiation • expressed in rapidly proliferating and dominant human PCa cell populations • promotes tumorigenicity, accelerates tumor growth, and migration, and enhances drug resistance	• CIC-TEX • EpCAM-CLDN-7 complex	(47–49)
claudin-11	• expression is inhibited in the occurrence of nerve invasion • Increased expression inhibits development of GC	• MALAT • inhibition of miR-135b with lncRNA PCAT18, promotes the expression of claudin-11	(26, 50, 51)
claudin-12	• poor prognostic factor	• SRSF1, LINC00857, and ZNF460	(52, 53)
claudin-18	• no specific staining detected in the normal pancreatic tissue, which was abnormally activated during the malignant transformation of pancreatic cells	• PKCd, PKCe, and PKCa • TPA and T/EBP/NKX2.1 • therapeutic target of Claudiximab (Zolbetuximab)	(54–57)
claudin-23	• induced isolation of weakly invasive and migratory PCa cells (PC-1)	• addition of a dissociation factor conditioned medium significantly reduced the expression of claudin-23 mRNA and protein in PC-1 cells	(7, 58)

ZEB1, zinc finger E-box binding homeobox 1.

## 4 Expression of CLDNs in PCa

Different claudin species have differential distribution and expression in the pancreas and PCa, and some claudin molecules have clinical significance. In this paper, we discuss the different types of claudins reported in PCa with a focus on research trends and clinical applications (54).

### 4.1 Claudin-18.2

#### 4.1.1 Distribution and regulation of claudin-18.2

Claudin-18 was first identified and reported as a new downstream target gene of the thyroid-specific enhancer-binding protein/NK2 Homeobox 1 (T/EBP/NKX2.1) homologous domain transcription factor. Claudin-18 is spliced into two specific isoforms, in lung and gastric tissues, with selective splicing of exons 1a and 1b forming claudin-18.1 and claudin-18.2, respectively. Claudin-18.1 is mainly expressed in normal lung tissue and LC cells, while claudin-18.2 transcript is restricted to gastric tissues under normal conditions and is expressed in the cell membrane under pathological conditions in cancerous tissues, such as GC, PCa, and esophageal cancer (18). Treatment with the protein kinase C (n-PKC) activator, 12-O-tetradecanoylphorbol 13-acetate (TPA), significantly induced claudin-18.2 mRNA expression in all the PCa cell lines and human telomerase reverse transcriptase (hTERT)-human pancreatic ductal epithelial (HPDE) cells in the pancreas. Additionally, claudin-18.2 protein expression significantly increased in highly or moderately differentiated human PCa HPAF-II, HPAC, and hTERT-HPDE cells. TPA-induced upregulation of claudin-18 in human PCa cell lines was blocked by PKCd, PKCe, and PKCa inhibitors and in hTERT-HPDE cells was blocked by PKCa, PKCu, and PKCd inhibitors. In addition, treatment with foponol 12-myristate 13-acetate (PMA) yielded similar results, suggesting that activation of the PKC pathway may be involved in claudin-18 expression associated with pancreatic carcinogenesis. yielded similar results, suggesting that activation of the PKC pathway may be involved in claudin-18 expression associated with pancreatic carcinogenesis. The coding sequence of the claudin-18 gene, and treatment with the demethylating agent, 5-azodeoxycytidine, enhanced the TPA-induced upregulation of claudin-18 in HPAF-II and HPAC, but not in hTERT-HPDE cells. This suggests that in human PCa cells, Claudin-18 is mainly regulated at the transcriptional level through specific PKC signaling pathways and modified by DNA methylation, involved in tumor differentiation and migration (55). Sequence analysis revealed that several other shared sequences, GATA and SOX transcription factors, are located in the proximal region of the human *CLDN*18a2 promoter and together regulate tumor differentiation and migration. The interaction between different cell-linked proteins has been demonstrated in human cancer cells, and the expression of integrin αvβ5 and claudin-18.2 in cancer tissues showed a significant positive correlation, which could be used to investigate the link between these two potential antibody targets to suggest new targeted therapeutic options. Claudin-18 has also been shown to interact directly with the EGFR pathway that promotes cell migration and proliferation. Epidermal growth factor (EGF) treatment and overexpression of RAS oncogenes induce claudin-18 expression through activation of extracellular signal-associated kinase (ERK)1/2. Furthermore, enhanced claudin-18 expression activated ERK1/2. Claudin-18 provides evidence for a role of claudin-18.2 in the oncogenic properties of cancer cells by regulating EGFR/ERK signaling (59). These results suggest the diagnostic and therapeutic potential of claudin-18.2 in PCa (56, 60, 61), supporting its use as a candidate target for the development of therapeutic antibodies (20, 57).

Claudin-18.2 is not detected in normal pancreatic tissues and is aberrantly activated during the malignant transformation of pancreatic cells (3, 34, 62). Regardless of the histological grade, the positive expression of claudin-18.2 in patients with primary PDAC was 94.6%, with 94% of the tumor cells exhibiting high claudin-18.2 expression in IHC staining (63). Additionally, Claudin is abnormally expressed in TPA-transfected hTERT-HPDE cells. Furthermore, normal HPDE cells receive stimulation to express claudin under certain circumstances, indicating that ectopic activation of claudin-18 is an early effect and PDAC usually has active dominant adherent proliferation and mesenchymal components, while tumor cells are rare. Intraepithelial neoplasia in PDAC includes pancreatic intraepithelial neoplasia (PanIN), mucinous cystic neoplasia (MCN), and intraductal papillary mucinous neoplasia (IPMN) (64), and *CLDN* gene expression was most significantly upregulated among the differentially expressed genes in IPMN (60, 65). In contrast, among specific types of PCa, such as pancreatic neuroendocrine tumors, only 20% of the patients were positive for claudin-18.2, with a strong staining intensity in all the positive patients (20). Notably, *CLDN18.2* expression was more prevalent and frequent than mucin 5AC (MUC5AC), an early marker for pancreatic ductal tumors, and claudin-18.2 had a specificity and sensitivity of 93% and 79%, respectively, for identifying the stomach, pancreas, and biliary tract as the primary tumor site, with the positive and negative predictive values of 76% and 94%, respectively. This suggests that claudin-18 can be a sensitive and specific marker for adenocarcinoma of the stomach and pancreatic bile duct (62), and claudin-18.2 is a valuable early marker of pancreatic bile duct tumors (60). Considering that *CLDN18.2* is expressed at comparable levels in primary and metastatic foci, the scope of clinical trials can be extended from patients with advanced PCa to those who develop metastatic PCa.

#### 4.1.2 Relationship between CLDN18.2 expression and pathological stages of PCa

*CLDN18.2* expression was significantly higher in stage III + IV PDAC than in stage I + II (P = 0.012), and its expression in stage IV was significantly higher than in the first three stages (P = 0.022), showing that *CLDN* expression is more pronounced in the later stages of disease progression. Activation and high expression of *CLDN18.2* are positively correlated with unfavorable prognostic factors for lymph node positivity. *CLDN* expression was also significantly higher in tissues with lymphatic infiltration compared to those without lymph node infiltration (P = 0.019), while nerve invasion was also associated with *CLDN18.2* expression as an independent factor (P = 0.006). As most PCa patients have metastases at the time of diagnosis, *CLDN18.2* expression in lymph node and liver metastasis samples from patients with advanced pancreatic tumors was comparable to or even higher than that of the primary focus (P = 0.022) (63). Furthermore, tumor cells show heterogeneous expression of *CLDN18.2*, with the phenomenon present in 9.7% of PDACs, and *CLDN18.2* expression in these tissues showed a diffused distribution, with a gradual downregulation of expression from the tumor margin to the deeper tumor.

*CLDN18.2* expression was statistically significant in terms of patient survival and prognosis. A stratified validation approach was used to find that the median survival of patients with positive *CLDN18.2* expression in stages III and IV decreased by 7 and 4 months compared to those with negative expression, and the median survival of patients with distant metastases was 7 vs 4 months (P = 0.024). This indicates that CLDN18.2-positive patients with advanced and distant metastases have a poor prognosis.

#### 4.1.3 Treatment of claudin-18.2 positive tumors

Currently, several claudin-18.2-targeting monoclonal antibodies (mAbs) are being used in clinical trials for PCa treatment. Zolbetuximab (IMAB362), a selective monoclonal immunoglobulin G1 antibody targeting claudin-18.2, binds specifically to tumor cells by binding to the first extracellular structural domain of the claudin-18.2 protein with high affinity and selectivity. It shows high antitumor activity, which is mediated by target-selective antibody-dependent cell-mediated cytotoxicity (ADCC), inducing complement-dependent cytotoxicity (CDC)-mediated lysis of claudin- 18.2-expressing tumor cells. Therefore, zolbetuximab shows unique cancer cell selectivity that allows for maximum anticancer potency and reduced toxicity, thus widening the therapeutic window and allowing for optimal dosing. A few trials have revealed that combination treatment with epirubicin-oxaliplatin-capecitabine (EOX) for first-line treatment is superior to treatment with EOX or zolbetuximab alone. Additionally, gemcitabine-induced overexpression of claudin-18.2 in PCa cells, enhanced zolbetuximab-induced ADCC (66), as evidenced by prolonged progression-free survival (PFS) and overall survival (OS) in PCa patients and no significant increase in nausea, vomiting, neutropenia, or anemia-related adverse events (AEs) compared with EOX treatment alone. This trial has entered the clinical phase II treatment of advanced G/GEJ and esophageal adenocarcinoma. Sahin et al. showed that claudin-18.2 positive patients with advanced G/GEJ and esophageal adenocarcinoma had significantly higher PFS (HR = 0.44, 0.29–0.67, 95% CI, P = 0.0005) and OS (HR = 0.55, 0.39– 0.77, 95% CI, P <0.005). The feasibility of zolbetuximab in the PCa treatment has been demonstrated in clinical trials, with the trials advancing to the volunteer recruitment phase; however, the clinical efficacy of zolbetuximab is yet to be improved (63, 64, 67, 68).

Zhong et al. found that the humanized variable domain of heavy chain of heavy chain antibodies (VHH) fused with human IgG1 Fc can also be a potential therapeutic candidate for PCa treatment, and *in vitro* experiments showed that it can induce ADCC and CDC in claudin-18.2 positive tumor cells. In the mouse xenotransplantation model, the anti-tumor effect of hu7v3-Fc was significantly higher than that of the reference anti-CLDN18.2 mAb, zobetuximab. In addition, *in vivo* bio-distribution using a zirconium-89 (89Zr)-labeled antibody showed that hu7v3-Fc has better tumor penetration and faster tumor uptake compared to zolbetuximab due to its smaller size and higher affinity (89Zr-hu7v3-Fc), suggesting its therapeutic potential for PCa treatment (69).

Anti-hCLDN18.2 ADC, CD3-bispecific and diabody, targeting a protein sequence conserved in rats, mice, and monkeys, were active against BxPC3/hCLDN18.2 (IC50 = 1.52, 2.03, and 0.86 nM, respectively) and KATO-III/hCLDN18.2 (IC50s of 1.60, 0.71, and 0.07 nM, respectively) were cytotoxic *in vitro* and inhibited pancreatic tumor growth. In a rat toxicity study, ADC tolerance was >10 mg/kg. In a preliminary evaluation of tolerance, anti-CLDN18.2 diabody (0.34 mg/kg) did not produce obvious signs of toxicity in the stomach of the NOD SCID gamma mice after 4 weeks of administration (70). Therefore, targeting claudin-18.2 in an ADC or bispecific manner may be an effective treatment for PCa. Another bispecific antibody, Q-1802, targets both programmed death ligand 1 (PD-L1) and claudin-18.2, thereby mediating ADC; in contrast, antibodies recognizing PD-L1 partially block programmed cell death protein 1 (PD-1) signaling and activate innate and adaptive immunity. Phase I clinical trials of this treatment have been initiated in patients with advanced solid tumors (NCT04856150). Anti-hCLDN18.2 ADC, CD3-targeting bispecific antibodies and diabody, targeting conserved protein sequences in rats, mice and monkeys, produce cytotoxicity *in vitro* and inhibit the growth of patient-derived xenograft tumors in the pancreas and stomach (71).

CT041 is an autologous chimeric antigen receptor (CAR)-T cell product candidate targeting claudin-18.2 for the treatment of patients with claudin-18.2-positive solid tumors. A study revealed that the subject patients developed ≥G3 hematologic toxicity, but dose-limiting toxicity (DLT) was not observed (72). Furthermore, the Phase II clinical trial of the drug (NCT04581473) is currently underway in patients with G/GEJ cancer and PCa, and the company is developing next-generation CAR-T cell therapy candidates targeting claudin-18.2, such as KJ-C1807 (CT048) for clinical trials in GC and PCa patients. In addition, mAb + monomethyl auristatin E (MMAE) drug conjugates targeting claudin-18.2 can effectively target tumor cells *via* anti-CLDN18.2 antibodies and trigger endocytosis, allowing the small molecule toxin MMAE to enter tumor cells to achieve antitumor effects. Preclinical studies have shown that SYSA1801 is biologically active, has a high safety profile, and has good clinical efficacy in GC and PCa (20).

Owing to its specific expression in PCa, claudin-18.2 has great potential as an early diagnostic and prognostic indicator of PCa (62). Therefore, the diagnostic, prognostic, and therapeutic potential of claudin-18.2 in PCa should be explored further (**Table 2**).

**Table 2:** Summary of clinical trials of drugs related to CLDN18.2.

NCT number	stage	Clinical trial	Cancer type	state
NCT03816163	II	IMAB362 + Nab-P + GEM	PCa	Recruitment
NCT03159819	–	CAR-CLD18T	GC and PCa	Recruitment
NCT03890198	I	LCAR-C182Acells	GC and PCa	Recruitment
NCT03874897	I	CAR-TCLDN18.2	solid tumor	Recruitment
NCT04404595	II	CAR-T (CT041)	GC and PCa	In progress
NCT05365581	–	ASP2138	G/GEJC and PCa	Recruitment
NCT05009966	–	SYSA1801	Advanced solid tumor, G/GEJC, and PCa	Recruitment

Data source: ClinicalTrails.gov.

### 4.2 Claudin-4

Claudin-4 and claudin-18 are highly and differentially expressed in PCa (37). Claudin-4 contributes to the function of anion channels/pores, the transepithelial resistance (TER) was enhanced in a claudin-specific manner. Claudin-4 is highly expressed in pancreaticobiliary ductal PCa (p = 0.015), PanIN, IPMN, MCN, and the major precursor lesions of pancreatic ductal adenocarcinoma (73), and its expression is correlated with the histological tumor grade of IPMN and MCN (11, 23, 34, 57). Similar to claudin-18.2, claudin-4 is also highly positive in liver metastases from PCa (74).

Patrick Michl et al. demonstrated that claudin-4 is a potent inhibitor of the aggressive and metastatic phenotype of PCa cells and a target of transforming growth factor (TGF)-β and the Ras/Raf/Erk pathway. Furthermore, proinvasive transforming factor-β downregulates claudin-4 in PANC-1 cells. Specific inhibitors of dominant negative Ras and downstream effectors of mitogen-activated protein/Erk and phosphatidylinositol 3’-kinase inhibit Ras signaling, thereby decreasing claudin-4 expression and in GC, which further increases PI3K and Akt phosphorylation, thus promoting tumor cell proliferation, migration, invasion, and tumorigenesis, a mechanism that may be similar in PCa (38, 39, 75). In studies of gastric cancer, claudin-4 expression was found to be associated with bivalent histone modifications, and decreased inhibitory histone methylation as well as increased active histone methylation was associated with claudin-4 overexpression in gastric cancer cells. Histone methylation and acetylation are required for claudin-4 alterations. The high expression of claudin-4 in pancreatic cancer needs to be further investigated and validated (76).

In the absence of TGF-β, DEC1 siRNA upregulated claudin-4, claudin-7, and E-cadherin expression and downregulated N-cadherin expression, consequently upregulating claudin-1 and claudin-4 (77). Tsutsumi et al. demonstrated that increased *CLDN4* mRNA expression was significantly associated with improved prognosis of PDAC patients and that reduced claudin-4 expression in endothelial cells reduced paracellular resistance and promoted the invasion of epithelial cancer cells through the endothelial cell layer, similar to the mechanism of E-cadherin (78). Histopathological examination revealed that the incidence of lymphovascular (ly), vascular (v), and neural (ne) invasion was higher in the low claudin-4 expression group than in the high claudin-4 protein expression group (P = 0.0014, 0.0133, and 0.0205, respectively) and overexpression of epithelial claudin-4 reduced the invasive and metastatic potential of the PCa cells *in vitro* (75). Research have shown that, median survival of patients with high claudin-4 expression was 63.0 months, compared with 14.7 months for patients with low claudin-4 expression (P = 0.0067). Immunohistochemistry (IHC) analysis revealed that *CLDN4* mRNA expression was significantly correlated with claudin-4 protein expression (P = 0.0168), indicating that increased expression of *CLDN4* mRNA can predict a better prognosis of PCa (27). Furthermore, claudin-4 is negatively expressed in reactive mesothelial cells and mesothelioma, differentiated from PCa, while it is significantly positively expressed in plasma membrane metastases and primary carcinoma of PCa. Therefore, claudin-4 can be used as an IHC reagent to exclude mesothelioma diagnosis (79). These results suggest that claudin-4 has great potential as a novel diagnostic biomarker for PCa (80).

Endoscopic ultrasound-guided fine-needle aspiration is a specific and effective marker for the differential diagnosis of pancreatic ductal carcinoma from gastrointestinal contamination and benign pancreatic ductal epithelium (81). claudin-4 co-localization *in vitro* and *in vivo* provides a method for early detection imaging diagnostic method for PCa (82). Claudin-4 also has great potential for targeted delivery and imaging, designed to label PCa using quantum dots (QD), as a sensitive optical contrast agent, and to image PCa by using anti-CLDN4 as a targeting ligand. Mintai P et al. developed a carrier protein-based PCa detection platform where any antibody and Ni-NTA-functionalized nanoparticles can bind the target protein *via* protein G and 6*His-tag, respectively; the carriers are modified by the desferrin gene to form a unique probe, which diagnoses PCa cells by detecting claudin-4 (83). The 18F-labeled CLDN-selective peptide, prepared by linking 5-(18F) fluoro-5-deoxyribose (5-(18F) FDR) to the oxime of the CLDN-selective peptide, provides a new imaging tool for PET-CT diagnosis of PCa. One of the 5-(18F) FDR clones27 with a molar activity of 4.0 GBq/µmol (for a 30 MBq tracer) was isolated at 98% radiochemical purity and 15% radiochemical yield (EOB) in 98 min. Therefore, these tracers contribute to the early diagnosis of pancreatic tumors (84). Thus, the specific expression of claudin-4 in PCa makes it a potential diagnostic target, which can be explored further.

CPE causes diarrhea and causes fluid accumulation in the intestines by altering the permeability of the intestinal epithelial membrane (85). It has an N-terminal cytotoxic region and a C-terminal receptor-binding region, and claudin-4 is sensitive to CPE-mediated cytolysis (86). mAbs can bind to ECL2 of the claudin-4 extracellular structure, which serves as a therapeutic target for tumors. Since CPE is a high-affinity receptor for this ring and causes cytotoxic, the C-CPE (receptor binding domain) was extracted and used in drug delivery and selective therapy of claudin-4-positive tumors (87–89).

A related study utilized translationally optimized CPE vectors (optCPE) for novel suicide pathways *in vitro* and *in vivo*, using cell line-derived and patient-derived PCa xenografts (CDX and PDX, respectively). This study demonstrated that apoptosis/necrosis signaling is activated *in vitro*, while necrosis and tumor cell killing is activated *in vivo* after implantation of the optCPE gene into PCa cells, which is mediated by pore formation. It was selectively toxic to claudin-3/4 overexpressing PCa cells. Meanwhile, downregulation of claudin-4 expression prevented CPE cytotoxicity (90), and optCPE non-viral *in vivo* intratumoral injection gene therapy showed targeted anti-tumor effects in different CDX and PDX PCa models, leading to reduced tumor cell viability and induction of tumor necrosis. optCPE treatment in combination with chemotherapy can further enhance tumor necrosis and CPE gene transfer. This approach is superior to other toxins used in cancer therapy because they act on more common non-tumor-specific targets (91). This combination therapy selectively acts on CLDN3/4 overexpressing tumors, *in vitro* and *in vivo*, thus improving PCa prognosis and contributing to the local control of PCa, especially in unresectable or refractory PCa (92). CPE is more cytotoxic to hypofractionated PCa than to highly differentiated PCa, and it is closely associated with the expression and localization of claudin-4 and barrier function (93).

The binding domain of claudin-4 and fusion can identify the abnormal localization of claudin in malignant tumors (86), and can be developed as a target for tumor treatment by using the fusion of cCPE and protein synthesis inhibitor (C-CPE-PSIF).The anti-CLDN18.2 mAb (5D12) binds to the second extracellular loop of claudin-4 and human-rat chimeric IgG1 of 5D12 activates the Fc gamma receptor IIIa, thus activating ADCC in CLDN4-expressing cells. It was found that anti-CLDN-4 mAbs significantly inhibited tumor growth in Dutch human CRC and GC mice without significant adverse effects, such as weight loss or liver and kidney damage, in PCa. Although human *in vivo* experimental effects remain to be proven in further experiments, the anti-propensity therapy of anti-CLDN-4 mAbs against claudin-4 targets is a promising treatment for PCa (94). Suzuki et al. isolated KM3900 (IgG2a), which specifically binds to claudin-4, from BXSB mice immunized with PCa cells. KM3900 recognizes and binds to the ECL2 of claudin-4 4, which can be detected in CLDN4-expressing PCa and OC cells. Mouse-human chimeric IgG1 (KM3934) induced dose-dependent ADCC and CDC *in vitro* and significantly inhibited tumor growth in ovarian cancer cells(MCAS or CFPAC-1) xenograft SCID mice *in vivo (* 87). CLDN4 serves to restrain pro-oncogenic signaling from EphA2 by limiting the activity of E-catenin and PI3K and preventing phosphorylation of EphA2 on S897 by AKT. This suggest that increases CLDN4 level and functional activity can inhibit the progression of cancer (95). These results suggest that claudin-4 is an effective target for cancer therapy and that anti-CLDN-4 targeting antibodies are promising therapeutic candidates.

Bioinformatics analysis suggests that claudin-4 may be regulated by FOX3 or USF2 and plays an important role in acute pancreatitis (96), and the variation in claudin-4 expression in PCa and acute pancreatitis needs to be further investigated.

### 4.3 Claudin-1

Claudin-1 is regulated by a variety of transcription factors, growth factors, and cytokines under normal physiological conditions, which maintains its tightly linked gating function (28). In pancreatic cancer, the expression of claudin-1 is increased, especially in metastatic tissues, there is a nuclear cytoplasmic membrane localization error. TNF-a upregulates the expression of claudin-1 in pancreatic cancer by concentration dependence, leading to accelerated tumor cell proliferation (28, 97).The region surrounding the CpG island of the *CLDN1* promoter controls transcription through methylation to alter epigenetic regulation. Furthermore, claudin-1 expression was negatively correlated with DNA methylation in short- and long-term survivor (GSE51820) (21) data analysis (98). ZEBI-regulated ZIP4 overexpression in PCa cells decreased the expression of ZO-1 and claudin-1, leading to phosphorylation of FAK and Paxillin, thereby promoting PCa invasion and migration (29). In contrast, ZEP4 knockdown increases claudin-1 expression, thereby reducing cancer cell invasion and metastasis. A study by Gurbuz showed that targeting 5-HT1B and 5-HT1D receptors downregulated ZEB1 and Snail family proteins while upregulating claudin-1 (30, 31). miR-193b expression resulted in increased expression of E-Cadherin and claudin-1 and decreased expression of Snail and TCF8/ZEB1 *via* the eEF2K and MAPK/Erk axes. Furthermore, miR-193b expression inhibited PDAC cell proliferation, migration, invasion, and EMT by suppressing the eEF2K/MAPK-Erk oncogenic axis (32). Additionally, the expression of claudin-1 gradually increases with the recovery of the disease while exhibit while exhibit opposite expression trend of cell-free DNA, in the mouse model of ulcerative colitis, the relationship between the content of the two is promising in the study of disease diagnosis indicators (99). In the future, targeted molecules related to claudin-1 can be prepared in a similar way to claudin-4 targeted drugs, and are expected to enter clinical practice (28).

### 4.4 Claudin-5

Claudin- 5 is widely distributed in the vascular endothelium, where it is associated with adhesion between arterial endothelial cells. The second extracellular structural domain of claudin-5 is capable of dimerizing *in vitro (* 100). Additionally, claudin- 5 is the most abundant TJ protein in the human blood-brain barrier and plays an important role in maintaining its integrity. Consequently, its dysfunction is associated with neurodegenerative diseases (Alzheimer’s disease), neuroinflammatory diseases (multiple sclerosis), and psychiatric disorders (depression and schizophrenia) (101), and the symptoms of these diseases can be eliminated to an extent by regulating claudin-5 expression.

Furthermore, claudin- 5 is involved in maintaining cell polarity and intercellular barriers (102). It is expressed in the vascular endothelium and some lymphatic vessels as well as in cells of many vascular and non-vascular tumors (40). It is expressed to a lesser extent in the subepithelial vessels of pancreatic plasmacytoma (SN) than in the interstitial vessels of SN (41). Claudin-5 is significantly positively expressed in pancreatic solid-pseudopapillary tumor cell membranes (SPN), but not in pancreatic neuroendocrine tumors (P-NET), and combined IHC detection of claudin-5 and intercellular adhesion molecule, CD99, is required for the diagnosis of SPN and P-NET (42).

### 4.5 Claudin-7

Claudin-7 is expressed in normal and malignant pancreatic tissue. It is expressed in the alveoli and ducts of the normal pancreas, and its expression is decreased in pancreatic ductal adenocarcinoma and is associated with the degree of differentiation of the tumor. In tumor cells, claudin-7 is present in two phases. Only GEM-located claudin-7 (palmitoylation) promotes tumor cell metastasis. Palmitoylated claudin-7 is involved in the development of drug resistance by inhibiting PTEN and subsequently activating the PI3K/Akt pathway (103). Because phosphorylated PTEN is not directed to GEM to compete with non-phosphorylated PTEN, association of cld7mPalm (claudin-7 has a mutated palmitoylation site) with the major PTEN phosphorylation kinase does not restore apoptosis resistance (104). Claudin-7 expression is negatively correlated with the glandular size of the tumor cells, and it is more pronounced in medium and large ductal tumors and well-differentiated tumors (47). Cancer-initiating cell-tumor exosomes (CIC-TEX) reprogram non-CIC into malignant tumors, and claudin-7, a biomarker for CIC, was recovered in gastrointestinal TEX. It was shown that individual cells delivering different microvesicles are particularly sensitive to claudin-7 and that claudin-7 located in the GEM is palmitoylated, which promotes the GEM-integrated plasma membrane and associated recruitment of signaling molecules. Non-palmitoylated claudin-7 recruits transport components, proteins involved in fatty acid metabolism, and TJ proteins into TEX. Claudin-7 also helps TEX recover selected miRNAs. Thus, well-localized claudin-7 affects CIC-TEX composition and claudin-7 may play a unique role in equipping CIC-TEX with transport proteins and regulatory molecules for lipid metabolism (48). CLDN7-associated epithelial cell adhesion molecules (EpCAM) are recruited to TEM, which support proliferation accompanied by sustained extracellular signaling regulating kinase-1/2 phosphorylation, anti-apoptotic protein upregulation, and drug resistance, but not EpCAM-mediated intercellular adhesion. This was only observed in EpCAM-CLDN-7-expressing cells where co-localization of claudin-7 with actin bundles may support enhanced motility. Additionally, the EpCAM-CLDN-7 complex strongly promotes tumorigenicity, accelerates tumor growth, and promotes ascites production and thymic metastasis formation. Therefore, high expression of the tumor marker, EpCAM, is often associated with poor prognosis (105). Claudin-7 functions through integrins to regulate cell proliferation and maintain epithelial cell attachment, and integrin β1 forms a complex of proteins that regulate cell growth and cell cycle progression, which in turn exerts control over tumor cell proliferation (106).

The solid papillary type (SPT) of PCa can be distinguished from the alveolar and neuroendocrine tumors by claudin-7 and claudin-5 immunoreactivity. Furthermore, SPT is positive for claudin-18.2and claudin-7 in a few cases, and pancreatic endocrine tumor (PET), acinar cell carcinoma(ACC), and pancreatoblastoma (PB) show strong expression of claudin-7 and a lack of claudin-5 expression (107).

Testicular pancreatic similar SPN is a recently reported entity that morphologically overlaps with the primary indolent mesenchymal tumor of the testis (PSRSTT), and its immune profile is positive for claudin-7. Based on the histological and genetic similarities found between pancreatic SPN and PSRSTT, it can be assumed that both tumors have the same pathogenesis; therefore, PSRSTT can be considered as a testicular analog of pancreatic SPN, and claudin-7 may play an important role in the diagnosis of this disease (108).

DNA microarray analysis showed that claudin-7 was highly expressed in MIA PaCa-2-A cells, and claudin-7 knockdown in MIA-PaCa-2-A cells significantly inhibited pancreatic tumor proliferation, reduced expression of p-Erk1/2, and inhibited G1 cell cycle arrest. Thus, *CLDN7* may be expressed in rapidly proliferating and dominant cell populations in human PCa tissues and could be a novel molecular target for PCa treatment (49).

### 4.6 Claudin-12

Claudin-12 plays an oncogenic role in PCa cells and promotes its malignant phenotype. Long intergenic non-coding RNAs (LNC00857) was found to affect the phenotype of PAAD cells by targeting miR-150-5p to regulate claudin-12 expression and by recruiting serine/arginine-rich splicing factor 1 (SRSF1) to promote selective splicing (AS) targeting claudin-12. ZNF460-regulated LINC00857 upregulates claudin-12 expression by sponging miR-1505p and recruiting SRSF1 to promote PAAD cell progression (53).Interleukin (IL)-18 is important in inducing BC cell migration *via* the downregulation of claudin-12 (52). Additionally, Claudin-12 promotes the proliferation and migration of osteosarcoma cells (109). Recent studies in PCa showed that knockdown of claudin-12 inhibited the proliferation of PANC-1 cells and downregulation of claudin-12 limited cell migration and invasion, further demonstrating that claudin-12inhibited EMT of PANC-1 cells. It is valuable to target miR-150-5p to regulate claudin-12 expression investigate therapeutic targets to inhibit PAAD cell progression and miR-150-5p.

### 4.7 Claudin-23

Human *CLDN23* mRNA is expressed in germinal center B cells, placenta, stomach, and colon cancer. It is a candidate oncogene associated with intestinal-type GC (7). Wei Wang et al. found that the dissociation factors (DFs) isolated from the medium of highly invasive and metastatic PC-1.0 PCa cells induced dissociation of weakly invasive and migratory PCa cells (PC-1), and the addition of DF-conditioned medium significantly reduced *CLDN23* mRNA and protein expression in PC-1 cells. These results suggest that *CLDN23* is involved in the regulation of PCa cell isolation through changes in gene expression and intracellular localization. In addition, Claudin-23 expression may be associated with the activation of the MEK signaling pathway during PCa cell isolation. Therefore, Claudin-23 also acts as a signaling molecule by actively translocating from cell–cell adhesion sites to the nucleus while simultaneously disrupting the structure of TJs (58). In colorectal cancer (CRC) studies, Claudin-23 expression was found to be epigenetically regulated and the significant downregulation of claudin-23 expression in tissues was attributed to the occupation of the claudin-23 gene, which encodes a structural component of cell-cell adhesion, by the Enhancer of zeste 2 (EZH2) and significant silencing in CRC tissues. The balance between the claudin-23 motif EZH2 methyltransferase activity and claudin-23 expression in CRC tissues, H3 lysine 27 trimethylation (H3K27me3) and H3K4me2, may underlie the regulation of claudin-23 expression. Existing studies suggest that this down-regulation causes disruption of intercellular adhesion function, providing more oxygen and nutrients to the microenvironment in which cancer cells live and enhancing their ability to metastasize (110). Studies on claudin-23 regulation in pancreatic cancer are scarce, and research on claudin-23 in pancreatic cancer could contribute to new targets for molecular therapy to prevent invasion and metastasis of pancreatic cancer.

## 5 Conclusion

In PCa, the claudin family plays an important role in cell junctions and cell barriers. Different types of claudin proteins play an important role in the EMT progression of pancreatic malignant and benign tumors, tumor development, nerve infiltration, tissue infiltration, and metastatic implantation. The specific expression of claudin molecules and expression levels differ in different types of PCa and PCa stages, and differential expression of claudins is associated with disease progression. It is also related to many factors such as organ type, genetic confounding factors and environmental background (6). The mechanisms of claudin phosphorylation and palmitoylation regulation of several types of claudin proteins have been revealed, but further research is needed in pancreatic cancer, and studies on claudin and classical tumor suppressors are still scarce. For instance, claudin-18.2, claudin-4, and claudin-6 are differentially expressed in normal and PCa tissues. Additionally, claudin-5 and claudin-7 have great potential in the diagnosis and differentiation of precancerous lesions and benign pancreatic tumors. Clinical studies on claudin-18.2 for GC have achieved promising results, and the development of claudin-18.2 and claudin-4-targeted drugs and combination therapies for PCa are also expected to provide promising results in PCa diagnosis and treatment.

## Author contributions

All authors listed have made a substantial, direct, and intellectual contribution to the work and approved it for publication.
